# Role of Septins in Endothelial Cells and Platelets

**DOI:** 10.3389/fcell.2021.768409

**Published:** 2021-11-11

**Authors:** Katharina Neubauer, Barbara Zieger

**Affiliations:** Department of Pediatrics and Adolescent Medicine, Division of Pediatric Hematology and Oncology, Medical Center, Faculty of Medicine, University of Freiburg, Freiburg, Germany

**Keywords:** septins, platelets, endothelial cells, angiogenesis, cell-cell junction, exocytosis, hemostasis

## Abstract

Septins are conserved cytoskeletal GTP-binding proteins identified in almost all eukaryotes except higher plants. Mammalian septins comprise 13 family members with either ubiquitous or organ- and tissue-specific expression patterns. They form filamentous oligomers and complexes with other proteins to serve as diffusions barrier and/or multi-molecular scaffolds to function in a physiologically regulated manner. Diverse septins are highly expressed in endothelial cells and platelets, which play an important role in hemostasis, a process to prevent blood loss after vascular injury. Endothelial septins are involved in cellular processes such as exocytosis and in processes concerning organismal level, like angiogenesis. Septins are additionally found in endothelial cell-cell junctions where their presence is required to maintain the integrity of the barrier function of vascular endothelial monolayers. In platelets, septins are important for activation, degranulation, adhesion, and aggregation. They have been identified as mediators of distinct platelet functions and being essential in primary and secondary hemostatic processes. Septin-knockout mouse studies show the relevance of septins in several aspects of hemostasis. This is in line with reports that dysregulation of septins is clinically relevant in human bleeding disorders. The precise function of septins in the biology of endothelial cells and platelets remains poorly understood. The following mini-review highlights the current knowledge about the role of septin cytoskeleton in regulating critical functions in these two cell types.

## Introduction

Endothelial cells (ECs) form a continuous thin layer (endothelium), which lines the interior surface of blood and lymphatic vessels separating the lumen from the surrounding tissue (Baldwin and Thurston 2001). The structure of ECs varies depending on the tissue (Gomez-Salinero and Rafii 2018). Vascular endothelium was originally considered a simple passive barrier and is meanwhile known to be crucial to maintain vessel integrity. Thereby, endothelial cell functions are versatile and comprise mediating vascular tone, hemostasis, regulating transport from the blood to underlying cells and tissues, permeability, cellular adhesion, smooth muscle cell proliferation, angiogenesis, and vessel wall inflammation (Kruger-Genge et al., 2019). Crucial communication partners of the ECs are platelets, small anuclear cells. ECs and platelets interact and regulate their activities mutually via direct and indirect signaling (Ruggeri 2003). One of the platelet’s key functions is to support hemostasis, a process of plug formation at the site of vascular injury to stop excessive bleeding. As a result of exposure to physical or biochemical stimuli, the initial activation step is the adhesion of platelets to the subendothelial matrix via collagen-bound von Willebrand Factor (vWF) and platelet glycoprotein (GP)Ib-V-IX complex. The vWF in plasma is released from ECs and platelets. Consequently, platelets come into close proximity of the injured vessel allowing the interaction of glycoprotein (GP)VI, the major collagen receptor on platelet membranes, with collagen. GPVI binding to collagen leads to platelet activation including the secretion of their intracellular granules and activation of the fibrinogen receptor integrin α_IIb_β_3._ Fibrinogen binding to integrin α_IIb_β_3_ in turn induces platelet aggregation with each other to form a hemostatic plug resulting in vascular occlusion. In this process, the platelet cytoskeleton reorganizes to undergo a change in shape from disc-shaped into spheres with protruding filopodia and lamellipodia (Jurk and Kehrel 2005). Although endothelial cells and platelets are different cell types, they share several properties. Both cell types derive from a common bone marrow-derived progenitor cell (Choi 2002). Not only platelets but also ECs circulate to a small amount in peripheral blood (Lin et al., 2000). ECs and platelets rearrange their cytoskeleton to promote changes in cell shape. They have the ability to store bioactive substances in cytoplasmic granules and release their cargo by exocytosis. Some endothelial and platelet proteins are identical, such as vWF or the cell adhesion molecule P-selectin (Denis 2002) Septins are also highly expressed in both ECs and platelets.

Mammalian septins comprise 13 family members which are either ubiquitously expressed or restricted to specific tissues or cell types (Dolat et al., 2014). All known septins are composed of a highly conserved central GTP-binding region flanked by N- and C-terminal with variable length. Most septins exhibit a C-terminal coiled-coil region, which may be necessary for interactions with other septins or proteins (Fung et al., 2014). Septins display the capability to form heterocomplexes *in vivo* and *in vitro*. The first characterized complex was the hexamer SEPT7-6-2-2-6-7 (Sirajuddin et al., 2007), which can become an octamer by the inclusion of two Septin 9 molecules (Sandrock et al., 2011). These polymers form further nonpolar filaments (Bridges and Gladfelter 2015) that can assemble in various higher-order structures, such as rings and gauzes by lateral stacking and tandem annealing (Kinoshita 2003; Valadares et al., 2017). Moreover, they can bind to phosphoinositides of cell membranes via a N-terminal polybasic domain (Zhang et al., 1999). Septin filaments thus represent an important component of the cytoskeleton, among actin, microtubules, and intermediate filaments (Mostowy and Cossart 2012). Septin-based structures may rearrange and disassemble in cells under the control of diverse factors and covalent modifications; however, the exact mechanisms of assembly and disassembly remain elusive (Kinoshita 2003). Septins participate in a spectrum of cellular processes involving the rearrangement of cytoskeletal elements or the motility of cellular membranes, for instance cytokinesis, cell polarity, endo- and exocytosis, or apoptosis (Hall and Russell 2004). Septin cytoskeleton is known to interact with actin filaments and microtubules in different contexts (Mostowy and Cossart 2012; Mavrakis et al., 2014; Bezanilla et al., 2015) and to contribute to intracellular membrane-associated processes, such as endomembrane fusion or mitochondrial fission (Mostowy and Cossart 2012; Dolat and Spiliotis 2016; Pagliuso et al., 2016). The cytoskeleton is a dynamic network and maintains cell morphology and movement, but it also enables rapid signaling events (Janmey 1998). Cytoskeletal remodeling is an important event in both ECs and platelets. The role of actin and microtubules in this process is well-established, whereas the septin network’s involvement is less clear. This mini-review focuses on the impact of septins in ECs and platelets summarizing the current knowledge in this field.

## Septins in Endothelial Cells

### Endothelial Septin Expression

Diverse reports show that septins are expressed in ECs in a distinct tissue-dependent expression pattern. In the human eye, SEPT4, SEPT5, and SEPT8 have been detected in corneal ECs (Pache et al., 2005). In human umbical vein ECs (HUVECs), SEPT2, SEPT4, SEPT5, SEPT7, and SEPT11 have been shown to be expressed (Blaser et al., 2006; Bartsch et al., 2010). Colocalization with the cytoskeletal protein α-tubulin was detected for SEPT2, SEPT4, SEPT7, and SEPT11 *in vitro* but only SEPT2 and SEPT7 colocalize with actin filaments (Bartsch et al., 2010).

### Septins Involved in Endothelial Endo- and Exocytosis

In HUVECs, SEPT4 and SEPT11 have been shown to be colocalized with the vesicle-associated protein synaptobrevin 1 (VAMP1), which belongs to the SNARE (soluble *N*-ethylmaleimide-sensitive factor attachment protein receptor) protein family (Bartsch et al., 2010). Since exocytosis is mediated by SNAREs (Chen and Scheller 2001) some researchers hypothesize that septins may be involved in exocytotic processes. Furthermore, SEPT2, SEPT4, SEPT7, and SEPT11 colocalize with the endocytosis marker transferrin receptor seemingly in the plasma membrane and in endosomes suggesting that these septins play a role in endocytotic processes. However, the impact of septins in these mechanisms in ECs remains unexplored (Bartsch et al., 2010).

SEPT7 is known to be involved in the endocytosis of microbial pathogens. Infection with Candida albicans leads to rearranged endothelial actin filaments forming pseudopods to pull the organism into the EC. In response to the infection, SEPT7 interacts with the endothelial adhesion protein N-cadherin. This SEPT7/N-cadherin complex accumulates around C. albicans hyphae and is necessary for the maximum endocytosis of C. albicans (Phan et al., 2013).

### Role of Septins in Angiogenesis

SEPT7 is reportedly involved in angiogenesis in microvascular cardiac ECs (Liu et al., 2014). ECs are the main players in angiogenesis, forming new blood vessels from preexisting ones. The major driver of this process is the arrangement of ECs in tip and stalk cells. Tip cells form filopodia that invade the surrounding tissue, leading the path toward neo-vessel formation (Eilken and Adams 2010). Angiogenesis requires cell polarity and the highly coordinated morphogenesis of ECs, which involves the accurate coupling of different cytoskeletal networks within the EC. In this process, SEPT7 has been shown to form a complex with Borg5 (Binder of the Rho GTPase 5) and to colocalize with actomyosin fibers (Liu et al., 2014). In general, Borg proteins have been shown to interact directly with septins and thereby influence actin and septin cytoskeleton (Sheffield et al., 2003). Liu et al. discovered that SEPT7/Borg5 facilitate the positioning and organization of contractile actomyosin fibers above the nucleus of primary mouse cardiac ECs. Genetic deletion of Borg5 as well as SEPT7 knockdown resulted in the disruption of perinuclear actomyosin and diminished persistent directional migration. The authors suggest that the SEPT7/Borg5 complex controls actomyosin activity to ensure persistent directional migration and efficient microvascular angiogenesis. Borgs are effectors of the Rho family GTPase Cdc42. Septin/Borg interactions have been found to be inhibited by constitutively active Cdc42. Overexpression of Cdc42 cause a loss of septin filaments (Joberty et al., 2001). A recent study shows that Cdc42 controls the subcellular localization of septins between actin stress fibers and microtubules (Salameh et al., 2021).

Vessel branching and angiogenesis is facilitated by the presence of podosomes. These are specialized compartmentalized actin-rich cell-matrix contacts able to locally secrete proteases and remodel the extracellular matrix (Gimona et al., 2008). A recent study showed that some septins (SEPT2, SEPT6, SEPT7, and SEPT9) are expressed in podosomes and identified the septin cytoskeleton as a novel component of endothelial podosomes (Collins et al., 2020). Collins et al. reported that SEPT2 in ECs is required for podosomal matrix remodeling and EC invasion, suggesting SEPT2 mediates the formation of functional podosomes, thereby facilitating the initial step of angiogenic invasion. The authors demonstrated that also SEPT6 and SEPT7 are necessary for regulation of matrix degeneration by ECs, but not SEPT9 despite all these septins were found in endothelial podosomes. This highlights the different roles for individual septins in this process.

There is also evidence that septins play a role in oxidative stress, a process caused by an imbalance of nitric oxide (NO) and reactive oxygen species (ROS). Oxidative stress induces vascular endothelial injury and is a mediator and modulator of angiogenesis (Kim and Byzova 2014). SEPT4 has been identified as an oxidative stress factor, which can promote oxidative vascular endothelial damage by interacting with apoptosis-related protein PARP_1_. This has been shown by knock-down and over-expression of SEPT4 (Zhang et al., 2018). Furthermore, SEPT4 is a physiological substrate of the ubiquitin ligase WWP2 involved in oxidative stress vascular endothelial injury. WWP2 mediates the degeneration of SEPT4, which inhibits formation of the SEPT4/PARP_1_ complex to suppress endothelial damage and vascular remodeling (Zhang et al., 2020).

### Septins Required for Cell-Cell Junction Integrity

The endothelial barrier function relies on cell-cell junctions that comprise tight junctions, adherens junctions, desmosomes, and gap junctions. Kim and Cooper characterized septin filaments in ECs using a monolayer system of primary human dermal microvascular ECs (Kim and Cooper 2018). They found SEPT2 located at cell-junction membranes and forming curved structures. SEPT2 was enriched especially in regions of positive membrane curvature associated with actin-rich membrane protrusions. Loss of SEPT2 led to a disrupted VE-cadherin structure and membrane dynamics, assuming that septins promote cadherin-based cell junctions and regulate the integrity of the barrier function formed by endothelial monolayers. SEPT2 may function as mechanical support for the actin-rich protrusions known to be essential for the assembly and stability of cadherin-based cell junctions.

In another study, Kim and Cooper extended their analysis by questioning whether SEPT2 is required to regulate the organization of other cell-adhesion proteins, focusing on PECAM-1 (platelet endothelial cell adhesion molecule-1), nectin-2, afadin, TJP (the tight junction protein), and ZO-1 (zonula occludens-1) (Kim and Cooper 2021). SEPT2 filaments are normally localized at junctions and are linked to the membrane by direct interaction with PIP_2_ (phosphatidylinositol 4,5-bisphosphate). Indeed, the loss of SEPT2 at cell junctions leads to striking spatial disorganization of all these junctional proteins: thereby, the expression levels of these proteins were unaffected by the loss of SEPT2 except for nectin-2, whose expression was greatly increased. These results suggest that the junctional location of SEPT2 is required for the sound organization of junctional proteins (Kim and Cooper 2021).

## Septins in Platelets

### Septin Expression in Platelets

In platelets, SEPT2, SEPT4, SEPT5, SEPT6, SEPT7, SEPT8, SEPT9, and SEPT11 are expressed as indicated *via* immunofluorescent staining, Western Blot analysis, or immunogold staining (Yagi et al., 1998; Zieger et al., 2000; Blaser et al., 2003; Blaser et al., 2004; Bartsch et al., 2010; Sandrock et al., 2011). There is no evidence to date for the expression of other septins. Platelet septins tend to form ring-shaped filaments (Martinez et al., 2006), which have been observed for SEPT5 and SEPT6 in suspensions of fixed human platelets. SEPT5 rings have also been detected in human fibrinogen-adhered platelets (Martinez et al., 2006). Sept8 displays rings in fibrinogen- and in collagen-adhered mouse platelets (Neubauer et al., 2021). Rings are the most often observed higher-order structure of septins in various organisms and cell-types. Such ring-shaped structures are often associated with other cytoskeletal networks, like actin and tubulin, or are located in discrete regions in the plasma membrane acting as diffusion barrier (Ewers et al., 2014) and/or cellular scaffold affecting various cellular functions, including cytokinesis (Gupta et al., 2018). In human platelets, SEPT5 and SEPT6 are located near the α-tubulin-rich platelet microtubule ring (Martinez et al., 2006). There is ample evidence that microtubules form a closed circular bundle running near the cell periphery, known as marginal band, which maintains the characteristic discoid shape of resting platelets (Kowit et al., 1988) suggesting that septin-rings may play a role in ensuring shape stability of resting platelets. Furthermore, SEPT5, SEPT6, and Sept8 display a punctate localization in the cytoplasm (Martinez et al., 2006; Neubauer et al., 2021). In platelets, septins demonstrate strong affinity to each other in forming complexes composed of multiple septin proteins (Sandrock et al., 2011).

### Platelet Septins Involved in Exocytosis

Platelet secretion of intracellular α-, δ-, and lysosomal granules is a form of regulated exocytosis, which is crucial for hemostasis, thrombosis, and inflammatory processes. In human platelets, SEPT4 and SEPT8 tend to be localized surrounding α-granules (Blaser et al., 2004) as well as SEPT5 (Dent et al., 2002). After platelet activation, SEPT4 and SEPT8 move to the platelet surface, suggesting a direct role in exocytosis (Blaser et al., 2004). Indeed, Sept8-deficient mouse platelets exhibit remarkably reduced α-granule secretion, but not of δ- or lysosomal granules (Neubauer et al., 2021). Transgenic overexpression of Sept5 in mice is associated with fewer and larger α-granules, indicating that Sept5 supports normally sized α-granules (Kato et al., 2004). In contrast, loss of Sept5 in mouse platelets leads to an enhanced release of δ-granules (serotonin) in response to subthreshold levels of agonists (Dent et al., 2002). A patient with homozygous co-deletion of SEPT5 and GPIbβ, a subunit of the platelet adhesion receptor (GP)Ib-V-IX, presented dramatically reduced platelet α-granule and slightly reduced δ-granule secretion, evidence not observed in patients with only a GPIbβ deletion but normal SEPT5 expression (Bartsch et al., 2011). Family members having a congenital pathogenic variant in the N-terminal region of the *SEPT9*-gene suffer from a mild δ-granule secretion defect (Neubauer et al., 2019). Bai et al. demonstrated that the N-terminal domain of SEPT9 binds and bundles microtubules by interacting with β-tubulin. In a cell line, a mutation in this region caused diminished intracellular microtubule bundling and impaired asymmetric neurite growth (Bai et al., 2013). During platelet activation, the marginal band needs to be re-arranged to ensure degranulation. The granules are concentrated in the platelet center and are surrounded by a smaller microtubule ring (White and Burris 1984). This close packaging is required for the fusion of granules not only with the plasma membrane but also between individual granules to guarantee a rapid secretion of the whole cargo. It had been hypothesized that the coiling of the marginal band, its compression through actomyosin contraction and the formation of a smaller microtubule ring leads to an accumulation of granules in the center of activated platelets (Sadoul 2015). Interestingly, Sept5 and Sept8 appear to have opposing roles, maybe because these two septins have been found to interact to individual factors of the exocytosis machinery. Platelets use soluble N-ethylmaleimide sensitive factor attachment protein receptor (SNARE)-mediated fusion of granules with the plasma membrane. Vesicular (v)-SNAREs and target (t)-SNAREs on the cell membrane form a protein complex to release granular cargo. The membrane fusion of α-granules and δ-granules requires different sets of SNAREs (Flaumenhaft 2003). SEPT5 has been hypothesized to inhibit the degranulation of δ-granules by binding the t-SNARE protein syntaxin 4 (Dent et al., 2002). In neurons, which share similarities with platelets in the control of neurotransmitter release and platelet granule secretion, respectively, Sept8 has been suggested to control the formation of the v-SNARE-complex at the synapse and subsequent exocytosis via dynamic interactions with VAMP2 (vesicle-associated membrane protein 2) (Ito et al., 2009).

### Sept8 is Involved in Primary and Secondary Hemostasis

In addition to the degranulation described above, Sept8 is known to be involved in platelet functions in primary hemostasis through comprehensive analyses using a Sept8-knockout mouse model (Neubauer et al., 2021). Loss of Sept8 in mouse platelets caused a pronounced defect in activation, adherence, and aggregation. In detail: Sept8-deficient platelets displayed a reduction in activated integrin α_IIb_β_3_. Platelet activation is associated with the binding of fibrinogen to the major receptor integrin α_IIb_β_3_ at the platelet surface, which is crucial for platelet bridging and subsequent aggregation. In contrast to the reduced aggregation caused by the loss of Sept8, a Sept5 deletion leads to an enhanced aggregation with an agonist requiring a platelet-secretory response (Dent et al., 2002). Sept8-deficient platelets exhibited delayed spreading on fibrinogen, a process by which adherent platelets flatten at sites of vascular damage and expand their contact area by deforming of the plasma membrane (Neubauer et al., 2021). Reorganization of the cytoskeleton network is essential for all these processes and for degranulation. This entails disrupting cell-cell adhesions, cell scattering, and enhanced cell motility. Sept8 could thus contribute to this process by interacting with actin filaments, microtubules, and intermediate filaments (Mostowy and Cossart 2012). In addition to primary hemostasis, Sept8 is known to be involved in secondary hemostasis (coagulation) (Neubauer et al., 2021). This is a process that leads to the formation of a stable platelet plug by activated coagulation factors, specifically thrombin, which converts fibrinogen to fibrin. A Sept8-deletion in mouse platelets leads to reduced thrombin generation. Furthermore, Sept8-deficient platelets exhibit reduced exposure of the membrane phospholipid phosphatidylserine (PS), which is provided physiologically by activated platelets on their cell surface and is incorporated into the developing clot demonstrating disturbed procoagulant activity of Sept8-deficient platelets. These findings revealed Sept8 as a modulator of distinct platelet functions associated with primary and secondary hemostatic processes.

### Regulation of Septins in Stored Platelets

A proteome analysis of platelet concentrates, which are essential in transfusion therapy, found that SEPT2 was quantitatively altered during platelets storage; the researchers hypothesize that septins may play a dynamic role during storage (Thiele et al., 2007). In a further study, microRNA-223 was found to regulate SEPT2 and SEPT6 in stored platelets (Chattopadhyay et al., 2018). Since platelets are anucleated cells, downregulation via microRNAs is one of the possible posttranscriptional mechanisms acting in platelets. MicroRNA-223 forms a complex with Argonaute 2 (AGO2) protein, the catalytic component of RISC (RNA Induced Silencing Complex).

## Conclusion

Eight out of 13 septins are highly expressed in both ECs and platelets and are known to play important roles (Table 1). Many of their functions are associated with reorganizing the cytoskeleton network, such as angiogenesis in ECs, or degranulation and spreading during platelet hemostasis. In addition, septins can also be a part of structures like cell-cell junctions where they are essential for the integrity of endothelial monolayers. However, the molecular mechanisms of septin-function in both cell types require further investigation in the future.

**TABLE 1:** Summary table of known septin localization and functions in endothelial cells and platelets.

	Endothelial cells			Platelets		
	Subcellular localization and colocalization	Functions	References	Subcellular localization and colocalization	Functions	References
Septin 2	Actin filaments		Bartsch et al. (2010)			
	α-Tubulin		Bartsch et al. (2010)			
	Cell-junction membranes; PIP_2_	Assembly and stability of cadherin-based cell junctions	Kim and Cooper (2018)			
		Organization of junctional proteins	Kim and Cooper (2021)			
	Podosomes	Matrix degeneration/Angiogenesis	Collins et al. (2020)			
Septin 4	α-Tubulin		Bartsch et al. (2010)	Surrounding α-granules		Blaser et al. (2004)
	PARP_1_	Apoptosis	Zhang et al. (2018)			
	Transferrin receptor	Endocytosis	Bartsch et al. (2010)			
	VAMP1	Exocytosis	Bartsch et al. (2010)			
	WWP2	Oxidative stress	Zhang et al. (2020)			
Septin 5				Ring near marginal band		Martinez et al. (2006)
				Surrounding α-granules	Exocytosis (δ-granule secretion)	Dent et al. (2002)
				Syntaxin 4		Dent et al. (2002)
Septin 6	Actin filaments		Bartsch et al. (2010)	Ring near marginal band		Martinez et al. (2006)
	α-Tubulin		Bartsch et al. (2010)			
	Podosomes	Matrix degeneration/Angiogenesis	Collins et al. (2020)			
Septin 7	Actomyosin fibers	Angiogenesis	Liu et al. (2014)			
	Borg5	Angiogenesis	Liu et al. (2014)			
	N-cadherin	Endocytosis	Phan et al. (2013)			
	Podosomes	Matrix degeneration and angiogenic invasion	Collins et al. (2020)			
	Transferrin receptor	Endocytosis	Bartsch et al. (2010)			
Septin 8				Ring	Platelet activation Exocytosis (α-granule secretion) Aggregation Spreading PS exposure Thrombin generation	Neubauer et al. (2021)
				Surrounding α-granules		Blaser et al. (2004)
Septin 9	Podosomes		Collins et al. (2020)		Exocytosis (δ-granule secretion)	Neubauer et al. (2019)
Septin 11	α-Tubulin		Bartsch et al. (2010)			
	Tansferrin receptor	Endocytosis	Bartsch et al. (2010)			
	VAMP1	Exocytosis	Bartsch et al. (2010)			

Abbreviations: PIP_2_, phosphatidylinositol 4,5-bisphosphate; VAMP1, vesicle-associated protein synaptobrevin 1; Borg5, Binder of the Rho GTPase 5; PARP_1_, apoptosis-related protein; PS, phosphatidylserine.
